# Welding X65 for Sour Service: Microstructural Evolution and Mechanical Degradation of Pulsed GMAW Joints in H_2_S Environments

**DOI:** 10.3390/ma19112306

**Published:** 2026-05-29

**Authors:** Rajesh Goswami, Jaykumar Vora, Basab Bhattacharya, Din Bandhu, K. Kumar, Najihah Mohd Tamyis

**Affiliations:** 1 Department of Mechanical Engineering, School of Technology, Pandit Deendayal Energy University, Raisan, Gandhinagar 382007, Gujarat, India; rajesh.gphd18@sot.pdpu.ac.in; 2 TechnipFMC Kuala Lumpur, TSLAW Tower, No. 39, Jalan Kamuning, Kuala Lumpur 55100, Malaysia; 3 Element Materials Technology, West Point Bizhub, 106 Tuas South Ave 2, Singapore 637158, Singapore; basab.bhattacharya@element.com; 4 Department of Mechanical Engineering, Galgotias University, Greater Noida 203201, Uttar Pradesh, India; 5 Malaysian Institute of Aviation Technology (UniKL MIAT), Universiti Kuala Lumpur, Lot 2891 Jalan Jenderam Hulu, Dengkil 43800, Selangor, Malaysia; najihah@unikl.edu.my

**Keywords:** X65 pipeline steel, pulsed gas metal arc welding (pGMAW), sour service, hydrogen embrittlement, fracture toughness (CTOD), electron backscatter diffraction (EBSD)

## Abstract

**Highlights:**

**What are the main findings?**

- pGMAW-welded X65 exhibits severe hydrogen embrittlement in sour service.
- Elongation drops 82% (22% in air to 4% after H_2_S exposure).
- Fracture toughness reduces by ~50% in both the weld centerline and HAZ.
- Fracture transitions from ductile dimples to quasi-cleavage with blistering.

**What are the implications of the main findings?**

- We provide critical baseline data for ECA of sour service pipelines.
- The findings mandate stringent NDE criteria for pGMAW welds in H_2_S environments.
- Weld metal requires special attention due to its higher embrittlement susceptibility.
- The results support the qualification of pGMAW for offshore X65 pipelines in sour applications.

**Abstract:**

This study investigates pulsed gas metal arc welding (pGMAW) of API 5L X65 pipeline steel for sour service applications where H_2_S exposure is anticipated. Mechanized pGMAW in the 5G downhill position was employed to fabricate girth welds using ER70S-6 filler wire with Ar-20%CO_2_ shielding. Comprehensive characterization, including optical microscopy, tensile testing, fractography, EBSD, and fracture toughness evaluation via SENT specimens, was conducted on specimens tested in both air and H_2_S-precharged sour conditions. Microstructural analysis revealed ferritic–pearlitic base metal, weld metal with acicular ferrite and bainitic constituents, and a transformed HAZ gradient. Tensile testing demonstrated severe hydrogen embrittlement in sour conditions, with elongation dropping from 22% in air to 4% after H_2_S exposure, accompanied by a transition from ductile cup–cone fracture to quasi-cleavage morphology. EBSD showed texture sharpening toward ⟨101⟩ fiber post-deformation, with a broader orientation spread under sour conditions, indicating heterogeneous strain localization. Fracture toughness testing revealed approximately a 50% reduction in CTOD values under sour exposure, with the weld centerline exhibiting greater degradation (0.50 mm to 0.27 mm) compared to the HAZ (0.92 mm to 0.47 mm). Fractography confirmed hydrogen-assisted cracking features, including shallow dimples, cleavage facets, and secondary cracking. These findings establish critical baseline data for engineering a critical assessment of pGMAW-welded X65 pipelines in sour service.

## 1. Introduction

Oil and natural gas are believed to be transported from subsea wellheads to offshore or coastal processing facilities most effectively and economically via pipelines. As a result, thousands of kilometers of offshore pipelines have been built throughout the world. To fulfill the ever-increasing demand for hydrocarbons, offshore oil–gas resource study and extraction have recently moved into deepwater and ultra-deepwater zones [1,2]. In a typical engineering project, several kilometers of offshore pipelines must be constructed on the sea floor in water between the depths of tens of meters and several thousand meters. Over the past few decades, a variety of installation methods for offshore pipes have been devised, taking into account sea conditions, water depth, pipe properties, economy, operability, and other aspects [3]. This provides a challenging condition for the designers, and fabrication is the matching of the mechanical and metallurgical properties of an entire pipeline assembly (including weld zones) to a sour environment which is highly corrosive. The S-lay method is one of the most popular methods because it can be used to lay pipes of various sizes in both shallow and deep waters [4,5].

Even in the challenging conditions, DNV L450 SFD SMLS, also known popularly as X65 steel, is one such steel that shows excellent weldability with GMAW and is also a candidate material for S-lay piping owing to its inherent mechanical and metallurgical properties. In order to achieve productivity, mechanized GMAW is successfully employed with X65 steel, and it shows promising results. This can be attributed to the fact that GMAW has the ability to generate a weld at a greater welding speed, with reduced heat input and ease of mechanization. Hence, the combination is growing in popularity in the fabrication of pipeline components [6,7]. The base metal is largely melted in GMAW using a solid wire electrode, and joint formation is aided by the base metal’s subsequent solidification. The welding pool is insulated from atmospheric contamination by the shielding gas, which also impacts the melting rate, weld bead morphology, and arc stability [8,9]. The performance of Argon with CO2 as a shielding gas is superior to Argon alone [10]. The base metal and filler wire are the main determinants of the shielding gas flow rate, which can range from 8 to 21 L/min [11]. An additional challenge is, most importantly, achieving the post-weld properties, such as fatigue growth and tensile strength, as well as microstructural features, especially in a sour environment [12]. Few studies have been reported wherein X65 is welded using a mechanized GMAW process and the resultant weld properties are provided. In one of the studies, Goswami et al. [13] qualified narrow-gap GMAW for S-lay offshore girth welds using ER70S-6 wire and 80% Ar-20% CO2 shielding. The study achieved acicular ferrite-dominated weld metal (WM) with fine HAZ grains, meeting the criteria for CTOD > 0.38 mm and an impact toughness of >100 J at −20 °C. This was attributed to low heat input (0.8–1.2 kJ/mm), which presented minimum coarse bainite. A similar study examined the submerged arc welding process, but benchmarked GMAW, wherein the weld metal showed acicular ferrite + bainite (270 HV hardness) and HAZ polygonal ferrite (220 HV). The toughness dropped to 53 J in the weld zone. The study recommended post-weld heat treatment (PWHT) for sour service [14]. Simionescu et al. [15] used the phased array Ultrasonic testing method to detect < 1 mm flaws in root/fill passes of the GMAW process on X65. The microstructure analysis revealed that refined ferrite–pearlite in the base metal transitions to bainite in the HAZ. This validates the high deposition rates (WFS 12–18 m/min) used in the study. One of the studies also examined the fatigue behavior of GMAW welds, and it was reported that the weld exhibits slower da/dN growth (Paris exponent m = 3.2) under R = 0.1 loading, which was attributed to compressive residual stresses and a uniform fusion line [16]. Safyari and Moshtaghi [17] attempted a Hybrid process for welding X65 steel, which yielded a 50% narrower HAZ (2 mm vs. 4 mm in pure GMAW) with finer acicular ferrite. A tensile strength of 620 MPa and an elongation of 25% were reported, which were superior to the conventional GMAW process. Yan et al. [18] assessed the corrosion fatigue behavior of X65 pipeline steel welded joints prepared using the CMT/GMAW process. The study reported that, in NACE A solution, GMAW welds showed a 40% lower crack growth rate than the base metal due to ferrite shielding. The study also reported hydrogen trapping at MnS inclusions. One of the studies analyzed GMAW welds in an X65MS sour service variant using optical/SEM and Charpy testing. The weld metal comprised acicular ferrite with granular bainite and side-plate ferrite, with hardness peaks at 270 HV. The HAZ showed refined polygonal ferrite (220 HV). The study also reported a low WM toughness (53 J at −40 °C) from oxide inclusions; the HAZ excels (>270 J) via recrystallization [14].

In another study, the EBW process was employed against GMAW for HSLA X65 pipeline repairs. EBW yielded a narrower HAZ (0.5 mm) with a bainitic–martensitic structure vs. GMAW’s 2–3 mm ferrite–bainite zone. The study also discussed GMAW limitations in hyperbaric subsea contexts, advocating for hybrids for future girth welds [19]. A pioneering study was carried out by Akselsen et al. [20] on remotely controlled GMAW for subsea pipeline repair/hot-tapping beyond diver depths (up to 2000 m). Welds overmatched BM strength (YS > 450 MPa); Charpy V-notch toughness reached 40 J min at −30 °C. The study reported the microstructure of acicular ferrite + bainite in LAS wire, austenitic in Ni filler. One of the studies compared pulsed GMAW, SAW and EBW on microalloyed X65 pipes (1.0 mm ER70S-6, Ar/CO2). GMAW heat input 0.8–1.2 kJ/mm yielded acicular ferrite in the weld metal and a narrow HAZ 1.5 mm wide with polygonal ferrite. A tensile strength of 480 MPa and a UTS of 580 MPa were achieved. Other properties, such as a toughness value of 80 J (−40 °C), and hardness of 240 HV, were reported. However, certain advantages, such as high deposition (15 m/h) and low Nitrogen (<100 ppm), which prevents cracking, were reported [21].

Based on the literature survey, mechanized GMAW has been established as a viable process for X65 pipeline welding. However, studies specifically addressing pulsed GMAW (pGMAW) for X65 in sour service applications remain scarce in the open domain. While Goswami et al. [13] demonstrated narrow-gap GMAW for offshore girth welds, and Zhang et al. [14] examined X65MS welded joints, no comprehensive investigation has systematically correlated pGMAW process parameters with the resulting microstructure, tensile properties, and fracture toughness under simulated sour service conditions. Furthermore, the combined application of EBSD texture analysis, CTOD fracture toughness testing, and fractography to assess hydrogen embrittlement susceptibility in pGMAW-welded X65 has not been reported.

This study addresses these research gaps by: (i) establishing a mechanized pGMAW procedure for an X65 line pipe in the 5G position; (ii) comprehensively characterizing the microstructural evolution across the base metal, weld metal, and HAZ; (iii) evaluating tensile properties and fracture toughness (CTOD) in both air and H_2_S-precharged sour conditions; and (iv) correlating the observed mechanical degradation with fractographic features and texture evolution using EBSD analysis. The findings provide critical baseline data for the engineering critical assessment (ECA) and qualification of pGMAW-welded X65 pipelines intended for sour service.

## 2. Materials and Methods

### 2.1. Base Material and Welding Variables

The starting material, DNV L450 SFD SMLS (X65) (Nippon Sumitomo Steel, Tokyo, Japan), was supplied in the form of seamless line pipes, each measuring 12 m in length. Two pipe sections were selected for welding. The pipes had an outer diameter of 16 in. (406.4 mm) and a wall thickness of 21.44 mm. The chemical composition of the as-received material is listed in Table 1.

**Figure 1:**
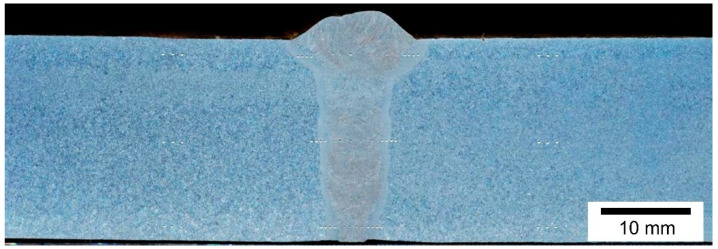
Macrostructure of the welded joint.

Welding of the line pipes was carried out using a mechanized pulsed gas metal arc welding (PGMAW) process, having a pulsating arc in the 5G (downhill) position. The polarity used was DCEP for five fill passes to fill the weld joint with an electrode sticking out 6–15 mm. A minimum preheat temperature of 60 °C was applied using propane torches and verified using contact thermocouples at three locations (weld start, mid-length, and end) on the pipe surface. The interpass temperature was strictly controlled between 60 °C and 250 °C throughout the welding process and monitored after each pass using an infrared thermometer. When the interpass temperature approached 250 °C, cooling was permitted in still air until the temperature dropped below 200 °C before depositing the subsequent pass. A mixture of 80% Argon + 20% CO_2_ was used as a shielding gas with a gas flow rate of 25–30 Lpm, and the filler wire of ER70S-6 (Lincoln Electric, Cleveland, OH, USA) had a diameter of 1 mm and a chemical composition shown in Table 1. The flow rate was maintained using a calibrated rotameter and verified at the gas nozzle before each weld pass to ensure consistent shielding coverage and prevent atmospheric contamination. Variable welding parameters are shown in Table 2. The optimal range of parameters was selected on the basis of some preliminary trials, prior knowledge, and an in-depth literature review. The welded macrostructure showing the weld profile is shown in Figure 1. Post-welding, the plates were subjected to mechanical and metallurgical testing as explained in subsequent sections. The welding was accomplished, and the testing for sour service and in air conditions was done separately after the welding.

### 2.2. Microstructural Testing

Microstructural analysis was performed on specimens extracted from the welded plate. The primary objective of the microstructural examination was to evaluate the microstructural modifications in the Base Material (BM), heat-affected zone (HAZ), and weld metal (WM) to correlate with the subsequent mechanical properties. The samples were extracted from a welded plate and then ground using progressively finer silicon carbide papers and subsequently polished using two different combinations of polishing cloths and abrasives to achieve a mirror finish. Finally, the specimens were etched using 3% Nital (3 mL nitric acid in 97 mL alcohol) to reveal the microstructural features. The samples were then observed under an optical microscope at different magnifications to reveal the micro-constituents.

### 2.3. Tensile Testing

The tensile properties of the weld joints were evaluated using dog bone-shaped tensile specimens. Tensile testing was carried out in accordance with ASTM E8M [22] at room temperature. Specimens with a 6 mm diameter were prepared as per ASTM E8 [22] requirements. A 25 mm gauge length extensometer was used to measure strain during the tests. Two categories of specimens were tested. The first set consisted of three samples of as-received weld metal specimens. The second set comprised three samples of weld metal tensile specimens pre-soaked for four days in a H_2_S environment (herewith referred to as a sour condition). The soaking was conducted using a gas mixture of 10% H_2_S balanced with N_2_, with EFC Solution A as the test medium. The gas mixture was continuously purged through the solution, and the specimens were fully submerged throughout the exposure period. The sample tests in air and sour conditions were conducted using strain rates of 0.015 and 0.005 mm/mm/min, respectively. The lower strain rate used for the sour-exposed specimens was selected to enhance sensitivity to hydrogen-assisted damage, since hydrogen embrittlement and stress–corrosion-related degradation are known to be strain rate-dependent and are often more clearly manifested under slow strain rate loading. The average values of three samples for each condition were taken for analysis purposes. Furthermore, to understand the tensile behavior, fractography analysis of the tested tensile sample was done using Scanning Electron Microscopy (SEM), wherein the fracture mode and the features were analyzed. To ensure consistency in fractographic comparison, SEM observations were performed using a predefined location protocol. Each fracture surface was first documented at a low magnification to identify the fracture origin, propagation region, and final overload region, followed by imaging at fixed magnifications from the same relative positions on all specimens. For cross-weld tensile specimens, fields were selected from comparable regions with respect to the weld centerline and adjacent HAZ, using identical SEM settings and field of view at each magnification. EBSD (electron backscatter diffraction), inverse pole figure (IPF) maps and pole figures were also generated for analyzing texture evolution, grain rotation, and twinning during tensile deformation. EBSD analysis was carried out on mechanically polished specimens extracted from the selected weld region before and after tensile testing. Final surface preparation was performed to obtain a deformation-free mirror finish suitable for diffraction pattern acquisition. EBSD measurements were conducted in a scanning electron microscope equipped with an EBSD detector, with the specimen tilted approximately 70 degrees to the incident electron beam. Orientation maps were acquired over selected representative areas using a fixed step size, and the resulting data were post-processed to generate inverse pole figure maps and pole figures for texture evaluation. Grain orientation spread, boundary character, and texture evolution were compared between air-tested and sour-exposed specimens. IPF maps visualize orientation changes and grain morphology, while pole figures plot the concentration of specific crystallographic planes, revealing preferred grain orientation (texture) relative to the tensile direction.

### 2.4. Fracture Toughness Testing

**Figure 2:**
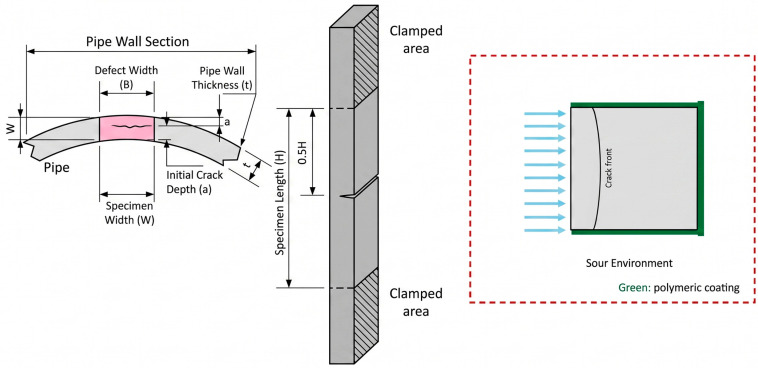
Schematic of SENT specimen for CTOD test and coating application for sour service testing.

Fracture toughness specimens were extracted from the weld joints and machined into Single Edge Notch Tension (SENT) specimens with B = 2W geometry, as shown in Figure 2. The nominal specimen dimensions were W = 13 mm (thickness), B = 26 mm (width), and 130 mm (length). The width (W) corresponded to the pipe wall thickness, with minimal machining to achieve a rectangular cross-section. An EDM notch was introduced from the inner diameter (ID) side and fatigue pre-cracked to achieve a target a/W ratio of approximately 0.35. Fracture toughness testing was conducted on specimens in both as-received and H_2_S pre-soaked conditions (referred to as sour condition). For testing in a sour environment, the fatigue pre-cracked SENT specimens were coated on all external surfaces, excluding the ID notch face, using a flexible ceramic coating to isolate the steel from the corrosive medium. The solution volume was maintained at 30 ± 10 mL/cm^2^ in accordance with NACE TM0177 [23]. Prior to testing, specimens were pre-charged in NACE Solution B under 99% H_2_S at 1 bar for four days, with the solution pH controlled at 4.8 to ensure hydrogen saturation. Mechanical testing was then performed in situ under continuous H_2_S charging using an environmental test chamber. Specimens were monotonically loaded under displacement control beyond maximum load at an initial K-rate of 1.5–2.0 N·mm^−3/2^·s^−1^, while recording load–CMOD responses. After testing, specimens were cooled in liquid Nitrogen and fractured to expose the crack surfaces. Initial and final crack lengths were measured, and CTOD values were calculated in accordance with BS 8571:2018. Metallographic and fractography examinations confirmed crack-tip location within 0.5 mm of the fusion line in the HAZ.

## 3. Results and Discussions

### 3.1. Microstructural Examination

**Figure 3:**
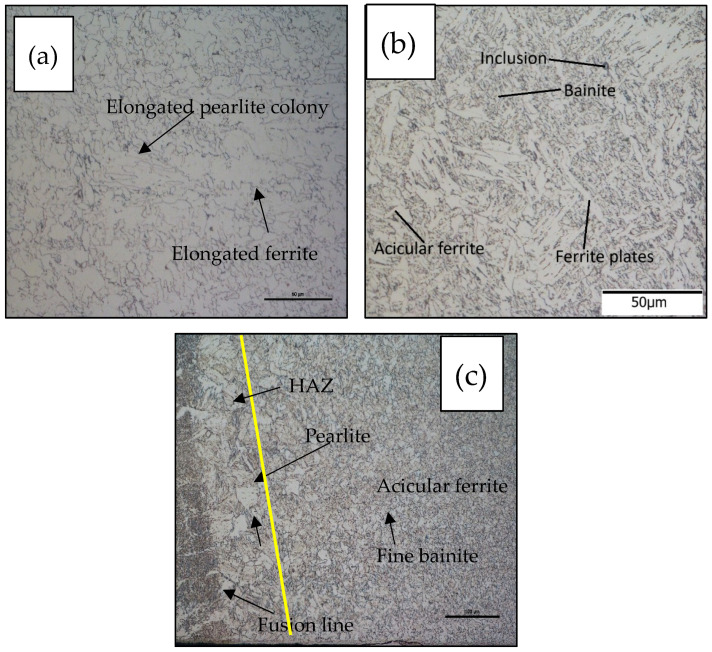
Microstructure images of (**a**) base metal, (**b**) weld metal, and (**c**) HAZ.

The microstructural images of the weldment are shown in Figure 3. Figure 3a reveals a predominantly ferritic–pearlitic structure of the BM, which is thermo-mechanically controlled processed (TMCP) pipeline steel. The ferrite phase appears as light regions, while the pearlite colonies are distributed as darker, elongated bands, indicating a controlled rolling process that promotes grain refinement and directional alignment. Multiple equiaxed ferrite grains interspersed with pearlite, suggesting a uniform and refined microstructure that enhances toughness and strength, can be observed in the image. A similar microstructure of BM was observed by Hashemi and Mohammadyani [24] in their study. The presence of elongated pearlite bands and slight banding indicates the influence of rolling direction, which is common in high-strength low-alloy steels designed for pipeline applications. Overall, the microstructure demonstrates a well-balanced combination of strength and ductility achieved through grain refinement and controlled cooling, making it suitable for demanding service conditions in oil and gas transportation.

Figure 3b demonstrates the microstructure of the WM area, which is consistent with the thermal cycle and solidification behavior of the PGMAW process. PGMAW provides controlled heat input and relatively high cooling rates, favoring acicular ferrite, which looks like fine, needle-like structures randomly oriented in carbon steel, including API 5L X65 [2]. The presence of a bainitic structure and ferrite side plates reflects localized variations in the cooling rate across the weld thickness. This mixed microstructure offers a balance of strength and toughness. However, the inclusion and bainitic laths can become potential hydrogen trapping sites in sour environments, explaining the reduction in fracture toughness observed during CTOD testing under sour environments [25]. Figure 3c demonstrates the HAZ microstructures of the weld metal, clearly showing a bright fusion line and exhibiting distinct phase transformations resulting from thermal cycles during welding. In the coarse-grained HAZ adjacent to the fusion line, the microstructure shows a mixture of bainitic and pearlitic phases formed due to rapid cooling from high peak temperatures. Moving away from the fusion boundary, the fine-grained HAZ consists predominantly of refined acicular ferrite and bainite, indicating lower peak temperatures and faster cooling rates that enhance toughness. The inter-critical HAZ, further away from the weld, displays partially transformed ferrite–pearlite regions, where some original base metal microstructure is retained alongside newly formed phases. Similar microstructural evolution in API 5L X65 weldments has also been reported in previous studies by King [26].

**Figure 4:**
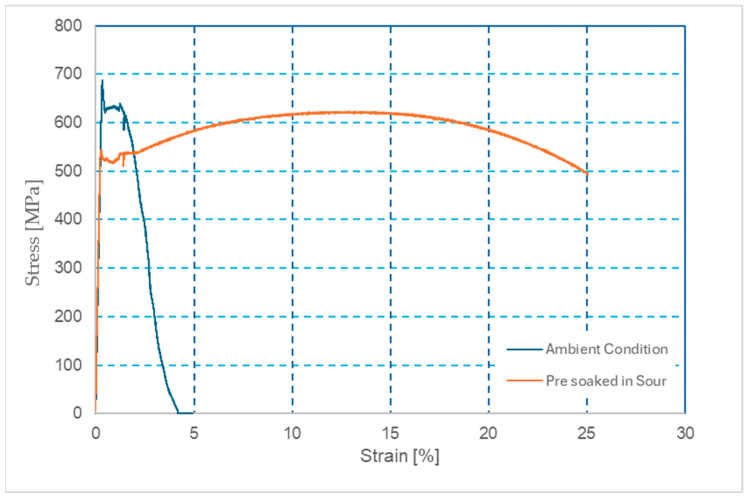
Engineering stress–strain curves obtained from tensile testing of the welded joint.

Tensile testing of the weld metal in air and sour conditions showed a clear difference in the mechanical properties of the material. The tensile testing results for both conditions are shown in Table 3, and the engineering stress–strain curves from the tensile testing are recorded and shown in Figure 4.

As depicted in Figure 4, the specimen tested in air exhibits typical ductile behavior. Following a brief yield point elongation, the material shows continuous strain hardening, with the tensile stress gradually increasing from the yield strength to the ultimate tensile strength (UTS). The UTS is recorded as approximately 600 MPa, followed by a gradual necking region extending up to about 23–24% of the total strain before the final fracture. The extended plastic deformation and smooth curvature of the stress–strain profile confirm the good ductility and stable strain-hardening mechanism of the welded joint when tested in an inert environment. In contrast, the specimen tested in the sour environment depicts significantly different behavior. Although the initially measured stress rises rapidly and reaches a slightly higher apparent UTS (approximately 630–680 MPa), the overall plastic deformation is severely reduced. After only 1–1.5% strain, a plateau region is observed, followed by a sharp and nearly continuous drop in tensile stress. Final fracture occurred at a much lower strain value of approximately 4–4.5%, indicating a substantial loss in ductility. This apparent increase in strength, coupled with a drastic reduction in elongation, is a characteristic of hydrogen-assisted embrittlement. Also, the same can be attributed to the combined effect of H_2_S as well as strain rates. In the sour environment, the observed mechanical degradation is consistent with hydrogen-assisted embrittlement mechanisms commonly reported for steels exposed to H_2_S-containing media. Under such conditions, hydrogen generated during surface reactions may enter the steel and interact with microstructural heterogeneities such as dislocations, grain boundaries, and inclusions, thereby promoting localized deformation, reducing uniform plasticity, and facilitating early crack initiation and propagation [27,28].

### 3.2. Fractography Analysis of Tensile Tested Specimen 

**Figure 5:**
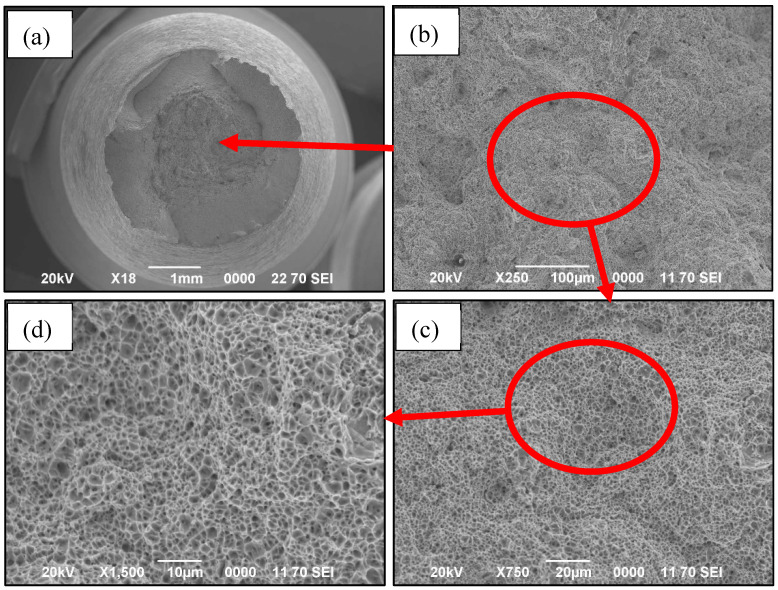
Fractography of the cross-weld tensile specimen tested in an air environment at room temperature. (**a**) Macro Image of fractured surface (**b**) Image at 250× Magnification (**c**) Image at 750× Magnification (**d**) Image at 1500× Magnification.

**Figure 6:**
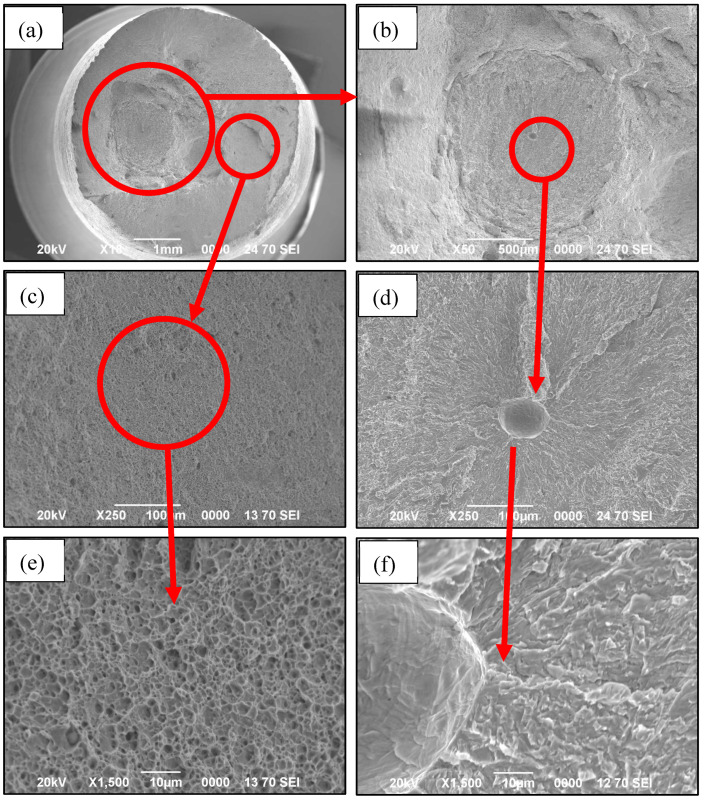
Fractography of the cross-weld tensile specimen tested in a sour environment at room temperature. (**a**) Macro Image of fractured surface (**b**) Image at 50× Magnification (**c**) Image at 250× Magnification of selected feature (**d**) Image at 250× Magnification of brittle fracture (**e**) Image at 1500× Magnification (**f**) Image at 1500× Magnification of brittle fracture.

The fracture surface analysis of the tested tensile specimen for both conditions is shown in Figure 5 and Figure 6. As shown in Figure 5a, the fracture surface clearly demonstrates a complete ductile failure characterized by a traditional cup and cone fracture with a distinct shear lip. Micro-voids appear across the surface, with void coalescence significantly contributing to crack propagation perpendicular to the axial force, as shown in Figure 5b–d, taken at different magnifications. Figure 5d demonstrates that a considerable amount of tearing/ripping of microstructures is seen as equiaxed dimples with clear evidence of micro-void coalescence, confirming a predominantly ductile fracture mechanism. In contrast, the fracture surface analysis shown in Figure 6 of the broken tensile specimen tested in a sour environment reveals a quasi-cleavage failure characteristic. This mixed-mode fracture displays features resembling brittle cleavage while still involving a limited degree of plastic deformation. The fracture surface is characterized by a comparatively narrow or tiny shear lip and a flat facet at the center, as seen in Figure 6a. The regions of shallow dimples and partially developed micro-voids can be seen in Figure 6b,c. In addition, distinct river patterns and feather-like markings, indicative of rapid crack propagation along preferred paths, are evident, as illustrated in Figure 6d,f, indicating a brittle fracture mechanism. These observations confirm the transition from a fully ductile fracture mode in air to a hydrogen-assisted, quasi-brittle fracture mechanism in the sour environment. Similar observations were made by Wang et al. [3] in their study on tensile testing of APLI 5L Gr. X65 steel in a sour environment. Figure 6e reveals that the dimple size becomes relatively smaller and shallower, indicating that the extent of the ductile failure mode is reduced after the hydrogen charging. Such a reduction in the size of dimples in sour service testing might be attributed to the fact that hydrogen decreases the maximum amount of stress and strain for the nucleation of voids. As a result of the decreased formation energy, void nucleation accelerates, resulting in the formation of smaller dimples on the fracture surface [29].

### 3.3. Texture and EBSD Pole Figure Study

**Figure 7:**
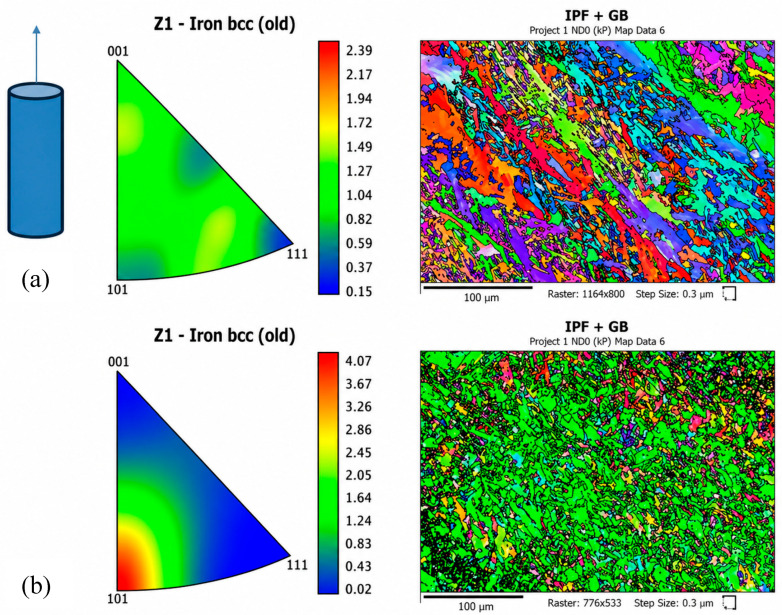
EBSD pole figure (**a**) before tensile test and (**b**) after tensile test in air.

The EBSD pole figures and IPF maps for the tensile testing specimen in air are shown in Figure 7. It can be seen that there is a clear deformation-induced texture sharpening and substructure evolution that is consistent with the as-welded microstructures. Before tensile loading—refer Figure 7a—the Z-direction inverse pole figure (IPFZ) indicates a weak, broadly distributed bcc texture (max intensity ≈ 2.4 m.r.d.) with orientations spread between ⟨001⟩, ⟨101⟩ and ⟨111⟩, reflecting the mixed as-welded constituents (coarse-/fine-grained HAZ with bainite, acicular ferrite and ferrite–pearlite). After tensile testing in air—refer Figure 7b—the IPFZ strengthens markedly (max intensity ≈ 4.1 m.r.d.) and the loading axis preferentially rotates toward the ⟨101⟩ fiber, in line with dominant 〈111〉 slip in bcc ferrite/bainite; the accompanying IPF maps show a fragmentation of the original lath/banded morphology into finer equiaxed subgrains and an increased fraction of low-angle boundaries, indicating dislocation accumulation and dynamic recovery. Correlating with the HAZ microstructure, the fine-grained regions containing acicular ferrite and bainite (more prevalent on the root side due to faster cooling) promote more uniform slip and stronger ⟨101⟩ fiber development. In contrast, the cap-side’s slightly coarser bainite exhibits weaker sharpening and more heterogeneous strain partitioning. Overall, the texture evolution toward ⟨101⟩ and the rise in boundary misorientations explain the observed balance of strength and ductility in air: strain hardening is provided by dislocation storage within bainitic/acicular ferrite laths, whereas recovery and subgrain formation in ferrite help sustain toughness—fully consistent with the HAZ phase gradients previously noted (coarse-grained HAZ: bainitic/pearlitic; fine-grained/intercritical HAZ: acicular ferrite + bainite + ferrite–pearlite).

**Figure 8:**
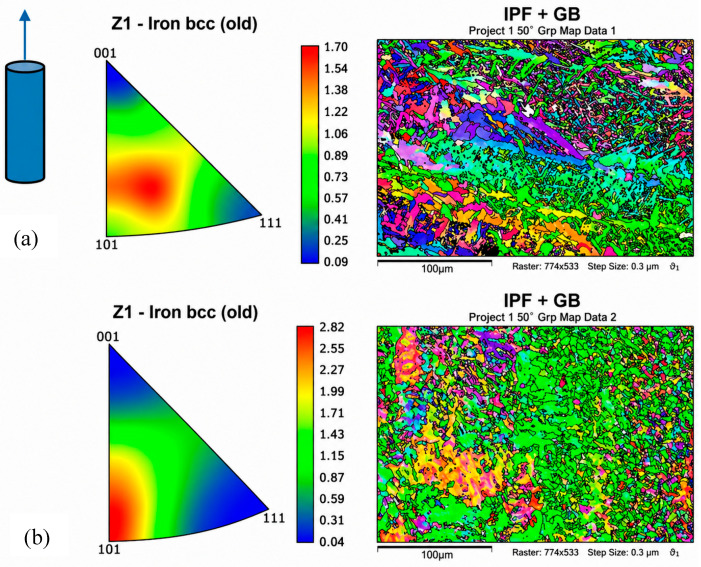
EBSD pole figure (**a**) before tensile test and (**b**) after tensile test, after soaking in a sour environment.

The EBSD pole figures and IPF maps for the tensile testing specimen in sour conditions are shown in Figure 8. It can be seen that significant texture and microstructural changes were present as compared to the air-exposed condition, consistent with the hydrogen-assisted degradation mechanisms described. Before tensile testing, the Z-direction pole figure shows a relatively weak bcc texture (max intensity ≈ 1.7 m.r.d.)—refer to Figure 8a—with orientations distributed between ⟨001⟩, ⟨101⟩, and ⟨111⟩, reflecting the original bainitic and ferritic phases but with evidence of local distortion and misorientation due to hydrogen ingress. The IPF map indicates a fragmentation of bainitic laths and more pronounced grain boundaries, correlating with carbide dissolution and localized micro-pitting observed in sour-exposed microstructures. After tensile testing, the texture sharpens toward the ⟨101⟩ fiber (max intensity ≈ 2.8 m.r.d.)—refer to Figure 8b. However, the overall orientation spread remains broader than in air, suggesting heterogeneous deformation and strain localization caused by hydrogen-induced embrittlement. The IPF map after deformation qualitatively suggests increased high-angle boundary density and subgrain formation, along with irregular morphology, consistent with hydrogen-assisted decohesion and microcrack initiation along bainitic lath boundaries and inclusions. These findings align with the microstructural observations: hydrogen diffusion and sulfide reactions weaken hard phases (coarse bainite) and promote decohesion, reducing ductility and increasing susceptibility to cracking under tensile load. The correlation between texture evolution and microstructural degradation highlights the combined effect of hydrogen trapping and phase instability on mechanical performance in sour service conditions.

### 3.4. Fracture Toughness Properties

**Figure 9:**
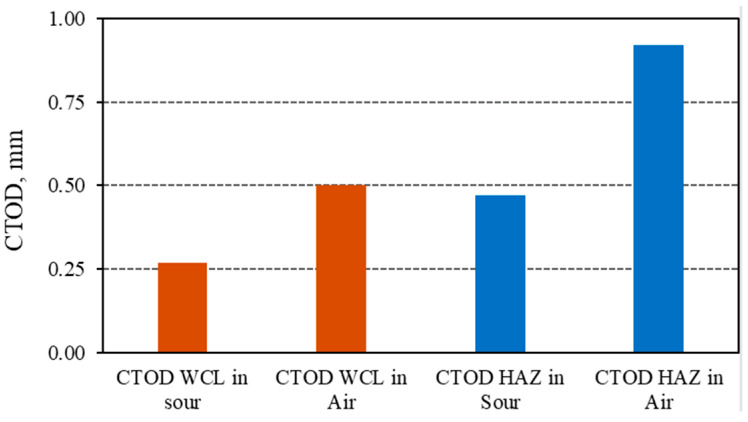
Comparison of CTOD test results obtained in air and sour environments.

The CTOD test results are illustrated in Figure 9. Fracture toughness was evaluated in terms of CTOD using SENT specimens in accordance with BS 8571:2018. During testing, the applied force and crack mouth opening displacement (CMOD) were continuously recorded. The CTOD values were determined using the BS 8571:2018 procedure for single-point fracture toughness evaluation based on the measured load–CMOD response, specimen dimensions, and crack size. The input parameters included specimen width, thickness, and initial and final crack length measured after test completion. The reported CTOD value corresponds to the selected event on the load–CMOD record in accordance with the standard. The results indicate a clear reduction in fracture toughness for specimens exposed to H_2_S compared to those tested in air. For the weld centerline (WCL), the CTOD value decreased from 0.50 mm in air to 0.27 mm after sour exposure, while for the HAZ the CTOD dropped from 0.92 mm to 0.47 mm, showing a large reduction in fracture toughness reflecting hydrogen influence. However, it shall be noted that the fracture toughness values obtained under H_2_S exposure conditions were still higher than those observed during the initial qualification testing of the welded coupon. These observations confirm that hydrogen ingress from H_2_S significantly impairs ductile fracture resistance, particularly in the weld metal region due to the presence of inclusions, acicular ferrite and bainite structure in the weld, which can be seen in Figure 3 and is discussed in the Microstructural Examination (Section 3.1). At the same time, HAZ shows relatively better toughness retention due to its finer microstructure. The fracture toughness results indicate an approximate 50% reduction in CTOD values for specimens tested in a sour environment compared with those tested in air. A similar trend of CTOD degradation under sour service conditions was also reported by Yuan et al. [30]. Based on this observed relationship, many industries perform CTOD testing in air and apply a conservative reduction factor of 30% to estimate fracture toughness under sour service conditions, thereby avoiding the need for direct testing in a sour environment.

### 3.5. Fractography Analysis of Fracture Toughness Specimen 

**Figure 10:**
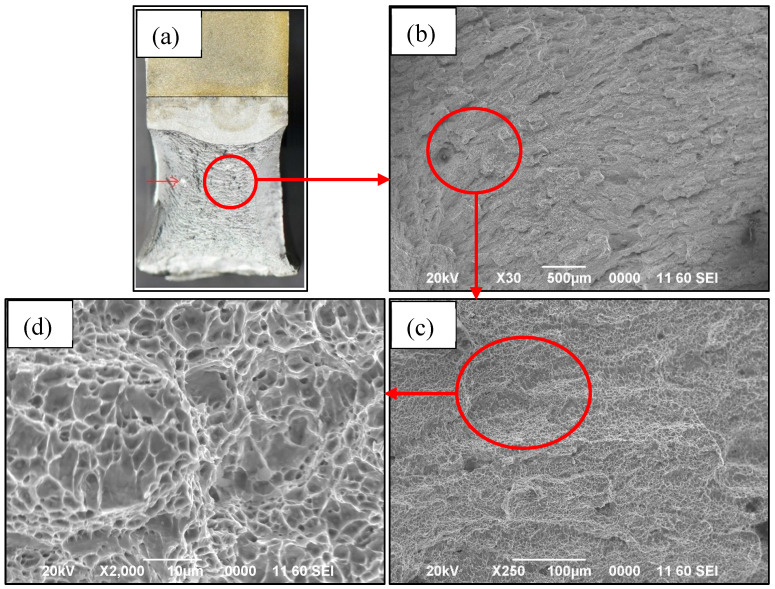
Fractography images of the fracture surface of the weld centerline (WCL) CTOD specimen tested in air. (**a**) Macro Image of fractured surface (**b**) Image at 30× Magnification (**c**) Image at 250× Magnification (**d**) Image at 2000× Magnification.

**Figure 11:**
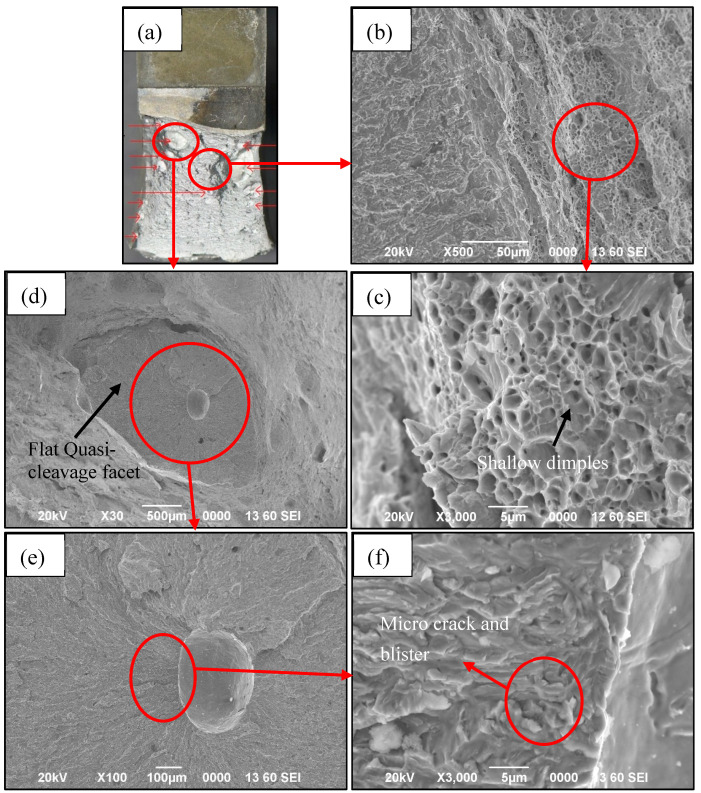
Fractography images of the fracture surface of the weld centerline (WCL) CTOD specimen tested in sour conditions. (**a**) Macro Image of fractured surface (**b**) Image at 500× Magnification (**c**) Image at 3000× Magnification (**d**) Image at 30× Magnification for brittle fracture (**e**) Image at 100× Magnification for brittle fracture (**f**) Image at 3000× Magnification for brittle fracture.

A fractography study was carried out on the fractured surfaces of CTOD-tested specimens after the completion of testing. Figure 10a shows the fracture surface of CTOD specimens extracted from the weld centerline (WCL) tested in air. A predominantly ductile fracture morphology with deep, equiaxed dimples formed by micro-void coalescence was confirmed by SEM fractography (see Figure 10b–d). The microstructural features at magnifications of 30×, 250× and 2000× revealed well-defined equiaxed dimples indicative of ductile fracture. This ductile response correlates with the WCL microstructure, which consists mainly of acicular ferrite interspersed with bainitic ferrite and ferrite side plates, as shown in Figure 3. The random orientation of acicular ferrite laths promotes crack path deflection and energy absorption, resulting in high fracture toughness in air. Figure 11a shows the macro-view of the fracture surface of the weld centerline (WCL) CTOD specimen tested in a sour environment, highlighting a mixed-mode fracture. Figure 11b,c show micrographs at 500× and 3000×, respectively, revealing a shallow dimpled morphology characteristic of ductile fracture.

In contrast, the images in Figure 11d–f captured at 30×, 100×, and 3000×, respectively, show well-defined cleavage facets, secondary cracking, and blister-like features indicative of brittle fracture behavior. Thus, the overall fractography analysis shows that the fracture features of the WCL specimen tested in the sour environment displayed a mixed-mode fracture surface with quasi-cleavage facets, microcracks, and blister-like features. These features indicate hydrogen-assisted cracking. Similar observations were noticed by Kyriakopoulou et al. [31] in their study. Microstructural analysis confirmed that inclusions and segregated regions within the acicular ferrite matrix act as hydrogen trapping sites, facilitating crack initiation and propagation under sour conditions [9,10]. These observations explain the significant reduction in CTOD values for WCL specimens tested in sour service compared to air.

**Figure 12:**
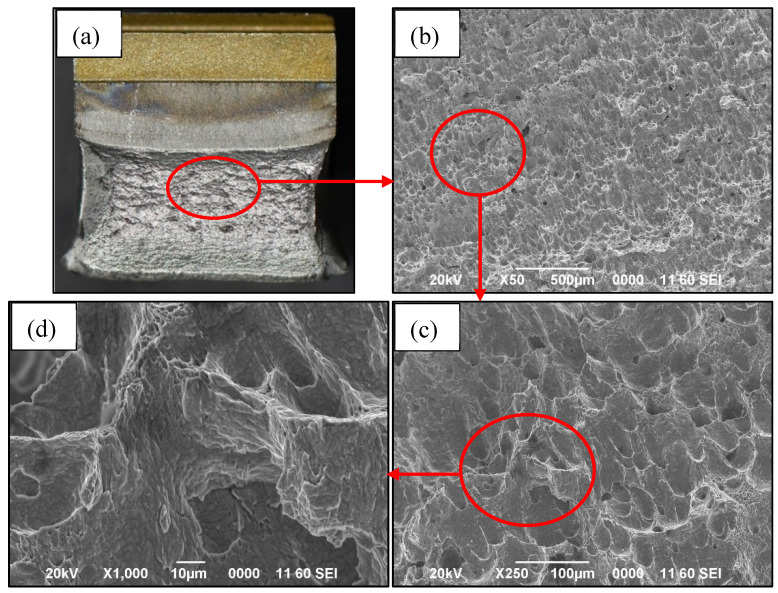
Fractography analysis of the fracture surface of the heat-affected zone (HAZ) CTOD specimen tested in an air environment. (**a**) Macro Image of fractured surface (**b**) Image at 50× Magnification (**c**) Image at 250× Magnification (**d**) Image at 1000× Magnification for brittle.

A similar study conducted on CTOD specimens from the heat-affected zone (HAZ) demonstrated a similar environmental influence but with less severity compared to WCL. Figure 12a shows the macro-view of the fracture surface of the heat-affected zone (HAZ) CTOD specimen tested in air, and the corresponding microstructural features at magnifications of 30×, 250×, and 1000× are shown in Figure 12b–d, respectively. The images reveal a well-defined equiaxed dimple indicative of ductile fracture. The air-tested HAZ specimen exhibited ductile fracture characteristics with well-developed dimples, consistent with micro-void coalescence as seen by Kyriakopoulou et al. [31] in their study. This behavior is supported by the HAZ microstructure, which comprises fine-grained acicular ferrite and bainitic structure, as shown in Figure 3, enhancing toughness and resistance to crack propagation.

**Figure 13:**
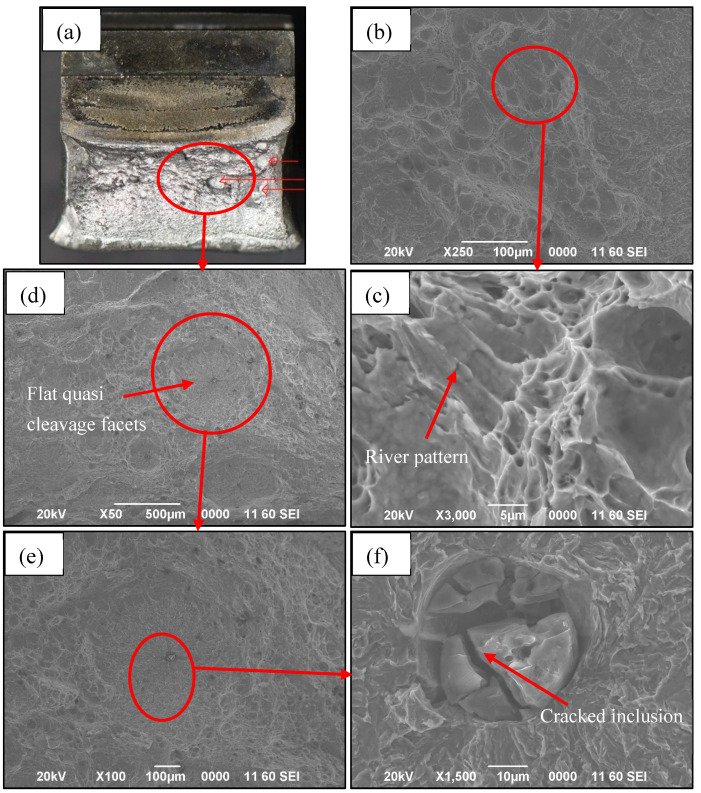
Fractography analysis of the fracture surface of the heat-affected zone (HAZ) CTOD specimen tested in a sour environment. (**a**) Macro Image of fractured surface (**b**) Image at 250× Magnification (**c**) Image at 3000× Magnification (**d**) Image at 50× Magnification for brittle fracture (**e**) Image at 100× Magnification for brittle fracture (**f**) Image at 1500× Magnification for brittle fracture.

In contrast, the sour-tested HAZ specimen showed localized brittle features, including flat quasi-cleavage regions, river patter, and cracked inclusion, as shown in Figure 13a. SEM fractography micrographs at magnifications of 250× and 3000×, shown in Figure 13b,c, respectively, reveal a shallow dimpled morphology characteristic of ductile fracture, illustrating the river pattern. Figure 13d–f, showing micrographs at magnifications of 50×, 100×, and 1500×, respectively, show well-defined cleavage facets, cracked inclusions, and features indicative of brittle fracture behavior. Furthermore, by correlating this with optical micrographs (Figure 4), it can be determined that coarse-grained ferrite and bainite act as hydrogen trapping sites, promoting localized decohesion and crack initiation under sour conditions [29,32]. Despite these effects, the CTOD reduction in HAZ was relatively minor compared to WCL, reflecting the beneficial influence of its finer microstructure. A comparison of WCL and HAZ fracture behavior highlights the critical role of the microstructure in determining sour service performance. Both regions exhibited ductile fracture in air and hydrogen-assisted embrittlement in sour conditions; however, the extent of degradation was greater in WCL. The acicular ferrite-rich WCL microstructure, while highly effective in promoting toughness in air, contains more inclusions and segregation zones that serve as hydrogen traps, leading to severe blistering and quasi-cleavage under sour exposure, similar to observations noticed by Chatzidouros et al. [33]. In contrast, the HAZ, with its finer ferrite and bainitic phases, demonstrated better resistance to hydrogen-induced cracking, as reflected by its smaller reduction in CTOD values. Overall, the correlation between fracture morphology, microstructural features, and CTOD results confirms that sour service significantly compromises fracture toughness, particularly in weld metal regions.

## 4. Conclusions

This study investigated the effect of sour service conditions on the mechanical performance and microstructural stability of pGMAW-welded API 5L X65 pipeline steel. The following conclusions are drawn:

- Microstructural Stability: Exposure to the sour environment promoted hydrogen-assisted microstructural degradation, with features consistent with possible sulfide-related effects, including localized surface damage and micro-pitting along bainitic lath boundaries and pearlite colonies. The fine-grained HAZ appeared to exhibit better resistance than the coarse-grained regions under the tested conditions.
- Tensile Properties: pGMAW welds exhibited severe hydrogen embrittlement under sour conditions and strain rate conditions, with elongation dropping from 22% in air to 4–4.5% after H_2_S exposure. Fracture morphology transitioned from ductile micro-void coalescence to quasi-cleavage features with river patterns and shallow dimples.
- Texture Evolution: EBSD analysis revealed deformation-induced texture sharpening toward the ⟨101⟩ fiber in both environments. Sour-exposed specimens qualitatively suggest broader orientation spread and increased high-angle boundary density, indicating heterogeneous deformation and enhanced susceptibility to hydrogen-assisted cracking.
- Fracture Toughness: CTOD values decreased by approximately 46% in the weld centerline (0.50 mm to 0.27 mm) and 49% in the HAZ (0.92 mm to 0.47 mm) under sour conditions. The weld metal showed greater susceptibility due to its acicular ferrite–bainite microstructure and inclusion content.
- Fractography: Both the WCL and HAZ exhibited ductile dimple fracture in air, transitioning to mixed-mode fracture with quasi-cleavage facets, secondary cracking, and blister-like features in a sour environment. HAZ demonstrated better toughness retention due to its finer grain structure.
- Novel Contribution: This study establishes a comprehensive correlation between pGMAW process parameters, resulting microstructures, and sour service performance of X65 pipeline welds, providing critical design data for offshore applications where H_2_S exposure is anticipated.

## Figures and Tables

**Table 1:** Chemical compositions of line pipe and welding filler wire used in this study.

Material	Alloying Elements (wt.%)									
	% C	% Si	% Mn	% P	% S	% Cr	% Mo	% V	% Ni	% Cu
DNV L450 SFD SMLS (X650)	0.07	0.13	1.45	0.01	0.002	0.12	0.06	0.06	0.09	0.09
ER70S-6	0.08	0.84	1.44	0.01	0.008	0.04	0.005	0.003	0.02	0.02

**Table 2:** Welding variables.

Welding Variables	Variables Range
Welding current (A)	131–245
Pulsing parameters	Base current (A)—65–138 Peak current (A)—325–480 Pulse width (Ms)—1.2–1.7
Wire feed speed (m/min)	5.5–11
Travel speed (cm/min)	30–75
Voltage (V)	17.5–26.3

**Table 3:** Tensile properties of pGMAW-welded X65 under air and sour conditions (mean ± standard deviation, *n* = 3).

Condition	YS 0.5% (MPa)	UTS (MPa)	Reduction in Area (%)	Elongation (%)
As received (air)	524 ± 8	606 ± 11	48 ± 3	22 ± 2
Sour condition	625 ± 15	687 ± 18	14 ± 2	4 ± 1
% change	+19%	+13%	−71%	−82%

## Data Availability

The original contributions presented in this study are included in the article. Further inquiries can be directed to the corresponding authors.

## Abbreviations

The following abbreviations are used in this manuscript:

API	American Petroleum Institute
ASTM	American Society for Testing and Materials
BM	Base Metal
BS	British Standard
CMT	Cold Metal Transfer
CO_2_	Carbon Dioxide
CTOD	Crack Tip Opening Displacement
DCEP	Direct Current Electrode Positive
DNV	Det Norske Veritas
EBW	Electron Beam Welding
EBSD	Electron Backscatter Diffraction
ECA	Engineering Critical Assessment
EDM	Electrical Discharge Machining
EFC	European Federation of Corrosion
GMAW	Gas Metal Arc Welding
H_2_S	Hydrogen Sulfide
HAZ	Heat-Affected Zone
HSLA	High-Strength Low-Alloy
HV	Vickers Hardness
ID	Inner Diameter
IPF	Inverse Pole Figure
KAM	Kernel Average Misorientation
LAS	Low-Alloy Steel
NACE	National Association of Corrosion Engineers
NDE	Non-Destructive Evaluation
pGMAW	Pulsed Gas Metal Arc Welding
PWHT	Post-Weld Heat Treatment
SAW	Submerged Arc Welding
SEM	Scanning Electron Microscopy
SENT	Single Edge Notch Tension
SMLS	Seamless
TMCP	Thermo-Mechanically Controlled Processed
UTS	Ultimate Tensile Strength
WCL	Weld Centerline
WM	Weld Metal
YS	Yield Strength
